# Associations of Serum Vitamin D Concentration with Cardiovascular Risk Factors and the Healthy Lifestyle Score

**DOI:** 10.3390/nu16010039

**Published:** 2023-12-21

**Authors:** Yerin Lee, Minju Kim, Inkyung Baik

**Affiliations:** Department of Foods and Nutrition, College of Science and Technology, Kookmin University, Seoul 02707, Republic of Korea; yherin@kookmin.ac.kr (Y.L.); km7985@kookmin.ac.kr (M.K.)

**Keywords:** vitamin D, heart disease risk factors, healthy lifestyle

## Abstract

Vitamin D status is reportedly associated with risk factors for cardiovascular disease (CVD), although conflicting data have been generated. The healthy lifestyle score (HLS) was formulated as a primary approach toward preventing CVD; however, data on the association between the HLS and vitamin D status remain insufficient. This study aimed to investigate the associations of CVD risk factors and the HLS with serum 25-hydroxyvitamin D concentration in adults who participated in a national survey. HLS components, including body mass index, smoking status, alcohol consumption, physical activity, and dietary pattern, as well as other risk factors, including diabetes mellitus (DM), hypertension (HTN), and dyslipidemia (DL), were fitted in multiple linear regression models to determine their association with vitamin D status. DM, HTN, and DL were inversely associated whereas a balanced dietary pattern, alcohol consumption, and physical activity were positively associated with serum vitamin D concentration (*p* < 0.01). Furthermore, a strong association was observed between the total HLS and serum vitamin D concentration (*p* for trend <0.01); the regression coefficient estimate (95% confidence interval) for the highest score was 1.41 (0.65, 2.17) (*p* < 0.01) compared with that for the lowest. These findings suggest that CVD risk factors and the HLS may reflect vitamin D status.

## 1. Introduction

Over one billion people worldwide have been estimated to suffer from vitamin D deficiency, which is defined based on serum 25-hydroxyvitamin D concentration [1]. Vitamin D plays a critical role in bone metabolism, and its deficiency increases the risk of rickets in children and that of osteoporosis and fractures in adults [2]. Furthermore, vitamin D deficiency is reportedly associated with the risk of cardiovascular disease (CVD) [3] and suggested as an emerging risk factor for CVD [4]. Accumulating epidemiological data have also revealed significant associations of low serum vitamin D concentrations with traditional risk factors for CVD, such as diabetes mellitus (DM), hypertension (HTN), dyslipidemia (DL) [5], smoking [6], and obesity [7]. Meanwhile, meta-analyses have suggested that vitamin D supplementation is significantly beneficial for preventing DM [8], HTN, and DL [9]; nevertheless, its direct effects on CVD events remain insignificant [10]. Based on these findings, vitamin D may influence CVD indirectly through its associations with CVD risk factors. Thus, further evidence regarding the association between CVD risk factors, especially lifestyle factors, and vitamin D status may be required to comprehend the link between vitamin D and CVD.

The healthy lifestyle score (HLS) is a comprehensive quantitative measure of healthy lifestyle factors, including optimal body mass index (BMI), nonsmoking, proper alcohol consumption, physical activity, and a balanced diet [11]. This score has been reported to be inversely associated with the risk of CVD, DM, and other chronic diseases [12,13,14]. Several studies involving adults from Western countries have reported associations of low serum vitamin D concentrations with smoking [6], obesity [7], low physical activity [15], and a certain dietary pattern [16]. In particular, a healthy dietary pattern based on Western diets, such as the Mediterranean diet, was found to be positively associated with serum vitamin D concentration [16]. Data regarding ethnicity-specific dietary patterns, especially those found among adults with non-European ethnicities, and HLSs involving such dietary patterns remain insufficient. One cohort study examined the association between the HLS and vitamin D status in older men in western Australia [17]; nonetheless, data on this association in Asian adults are considerably limited.

Therefore, the current study aimed to investigate the associations of serum 25-hydroxyvitamin D concentration with CVD risk factors and the HLS, including a dietary component that was defined based on a dietary pattern empirically derived from national survey data on Korean adults.

## 2. Participants and Methods

### 2.1. Study Participants

In this study, we utilized data from the Korean National Health and Nutrition Examination Survey (KNHANES), which is a cross-sectional national survey administered by the Korea Disease Control and Prevention Agency (KDCA). All KNHANES participants, who were noninstitutionalized South Korean citizens, provided informed consent [18,19]. All KNHANES data are publicly available (https://knhanes.kdca.go.kr/knhanes/ (accessed on 4 September 2023)). In particular, we selected KNHANES data collected during the 4th−5th survey period (2008–2012) because serum 25-hydroxyvitamin D levels were assayed during this specific period.

Among the 45,811 participants included in the stage 4–5 survey (n = 9744 in 2008, n = 10,533 in 2009, n = 8958 in 2010, n = 8518 in 2011, and n = 8058 in 2012), 15,576 were eligible according to the inclusion criteria (age 40–64 years). The exclusion criteria were participants whose serum vitamin D concentration was not assayed (n = 1508) or who were pregnant (n = 1). In addition, participants with missing data regarding DM, HTN, and/or DL diagnosis (n = 441), lifestyle factors, including BMI, smoking status, alcohol consumption status, and physical activity (n = 65), or other confounding variables (n = 6) and those with improper data on daily energy intake (<500 or >5000 kcal) (n = 1852) were further excluded. Thus, 11,703 participants (4744 men and 6959 women) were finally included for statistical analysis. The present study was approved by the Institutional Review Board of Kookmin University (approval number: KMU-202102-HR-260).

### 2.2. Serum 25-Hydroxyvitamin D

The outcome variable of our study was the serum 25-hydroxyvitamin D concentration, which was assayed by commercial laboratories. According to KNHANES reports [18,19], this biomarker was assayed using a radioimmunoassay kit (BioSource, Belgium or DiaSorin Inc., Stillwater, OK, USA).

### 2.3. Dietary Information

Dietary information was obtained using the 24 h recall method and organized into 20 food group variables by the KDCA. Based on a previous report [20], 18 food group variables were used in this study: “white rice”, “grains”, “potatoes/starch”, “noodles and dumplings”, “flour and bread”, “meat”, “seaweeds”, “seafood”, “eggs”, “beans”, “nuts”, “vegetables”, “kimchi”, “mushrooms”, “fruits”, “milk and dairy products”, “snacks and cakes”, and “fast foods”.

### 2.4. CVD Risk Factors and the HLS

DM, HTN, and DL diagnoses were defined based on (1) questionnaire responses regarding medical history and (2) health examination data on blood pressure (BP) measurements and blood profiles of glucose and lipid metabolism. DM was defined as a fasting glucose level ≥ 126 mg/dL or the use of diabetic medication or insulin injection; HTN as a high BP (systolic BP ≥ 140 mmHg and/or diastolic BP ≥ 90 mmHg) or the use of antihypertensive medication; and DL as a total cholesterol level ≥ 240 mg/dL, a high-density lipoprotein (HDL) cholesterol level < 40 mg/dL, or a triglyceride level ≥ 200 mg/dL. Information on lifestyle factors, including BMI, smoking status, alcohol consumption status, and regular physical activity, which was classified based on three questions regarding regular walking (at least five occasions/week for ≥30 min/occasion), moderate exercise (at least five occasions/week for ≥30 min/occasion), and vigorous exercise (at least three occasions/week for ≥20 min/occasion), was collected from questionnaire data. Using this lifestyle information, the HLS was calculated, and total scores ranged from 0 to 5 points. One point was assigned to each healthy lifestyle component according to the following criteria: BMI < 25 kg/m^2^; never smoked; average alcohol consumption of two or less drinks for men and one or less drinks for women; regular physical activity; and a balanced dietary pattern, which was derived from the factor analysis wherein factor scores were those greater than the median values.

### 2.5. Potential Confounding Variables

Information on potential confounding variables, including survey period, age, sex, residential district, household income level, educational level, occupational type, working hours, sun exposure time, average sleep duration, and use of dietary supplements (vitamins, minerals, and functional foods), was collected from questionnaire data. Additionally, total calorie intake, calculated using 24 h recall data, was considered a confounding variable.

### 2.6. Statistical Analysis

Because a complex stratified sampling design was applied in the KNHANES, proper statistical procedures considering sampling weight were used in all statistical tests. Descriptive statistics are presented as the mean ± standard error or percentage. Statistical differences between groups were tested using the Chi-square test and analysis of variance. To analyze the associations of serum vitamin D concentration with CVD risk factors and the HLS, linear regression analysis was performed to obtain regression coefficient estimates and their 95% confidence intervals (CIs). Potential confounding variables, including survey year (continuous), age (continuous), sex, residential district (three categories: special cities, metropolitan cities, and rural areas), household income level (two categories: lower-middle and high), educational level (two categories: middle school graduate and higher education), occupational type (four categories: office, service, manufacturing, and not employed), working hours (three categories: full-time, part-time, and others), sun exposure time (three categories: <5 and ≥5 h/day, and “no answer”), average sleep duration (four categories: <6, 6–7.9, 8–9.9, and ≥10 h/day), use of dietary supplements (two categories: “yes” and “no”), and total calorie intake (continuous), were incorporated into the multiple regression models. As exposures, BMI (four categories: <18.5, 18.5–22.9, 23.0–24.9, and ≥25.0 kg/m^2^), smoking status (two categories: never smoked, former and current smoker), alcohol consumption status (two categories: lifetime abstainer, former and current drinker), dietary pattern factor scores (quartiles), regular physical activity (two categories: “yes” and “no”), DM, HTN, or DL diagnosis (two categories: “yes” and “no”), and healthy lifestyle scores (five categories: 0–1, 2, 3, 4, and 5 points) were fitted in the analysis. These exposure variables were mutually fitted in the multiple models; in particular, BMI was fitted as a continuous variable.

To identify dietary patterns, factor analysis was conducted using dietary data on the 18 food groups. The number of dietary patterns was determined based on eigenvalues (≥1.3) and scree plots. Factor loading values for the food group variables were calculated. Dietary pattern factor scores were also calculated and assigned to each individual.

All statistical analyses were performed using the SAS software (version 9.4; SAS Institute Inc., Cary, NC, USA). Statistical significance was set at *p* < 0.05.

## 3. Results

### 3.1. Participants’ Dietary Patterns

Table 1 shows the dietary patterns derived from the factor analysis, with factor loading values for the food group variables. Based on the factor loading values, two types of dietary patterns were derived and labeled: “an imbalanced diet” and “a balanced diet”. An imbalanced diet was characterized by a high consumption of white rice and kimchi. In contrast, a balanced diet was typified by a high consumption of various food groups, including vegetables, eggs, meats, seafood, fruits, grains, flour and bread, and beans.

### 3.2. Participant Characteristics According to Serum Vitamin D Concentration

Participant characteristics were compared according to serum vitamin D concentration quartiles (Table 2). Participants with higher vitamin D concentrations were more likely to be older, be male, reside in rural areas, be less educated, be non-office workers or unemployed, be part-time workers, be smokers, be exposed to the sun, take dietary supplements, perform regular physical activity, have higher balanced diet factor scores, and consume alcoholic beverages.

### 3.3. Associations between CVD Risk Factors and Serum Vitamin D Concentration

Table 3 shows the associations of serum vitamin D concentration with BMI, smoking status, alcohol consumption status, dietary pattern, physical activity, and comorbidities. BMI, smoking, and an imbalanced diet were not associated with serum vitamin D concentration. However, DM, HTN, and DL were inversely associated (*p* < 0.05, *p* < 0.05, and *p* < 0.01, respectively), while a balanced dietary pattern (*p* for trend = 0.001), physical activity, especially regular walking (*p* < 0.001), and current alcohol consumption (*p* < 0.001) were positively associated with serum vitamin D concentration. In particular, the serum vitamin D concentration was highest for a balanced dietary pattern; the multiple regression coefficient estimate was 1.29 (95% CI: 0.83, 1.75) (*p* < 0.001) for the highest quartile of factor scores compared with that for lower quartiles.

### 3.4. Associations between the HLS and Serum Vitamin D Concentration

Table 4 shows the associations of serum vitamin D concentration with the HLS and its components. Similar to the results shown in Table 3, a balanced diet (*p* < 0.001), regular physical activity (*p* < 0.001), and alcohol consumption (*p* < 0.01) were positively associated with serum vitamin D concentration. Moreover, the total HLS was positively associated with serum vitamin D concentration (*p* for trend < 0.01). Compared with the lowest-score group, the highest-score group yielded a regression coefficient estimate of 1.41 (95% CI: 0.65; 2.17) (*p* for trend < 0.01).

## 4. Discussion

In this cross-sectional study based on national survey data, we investigated the associations of serum vitamin D concentration with CVD risk factors and the HLS, which incorporated BMI, smoking status, alcohol consumption status, physical activity, and dietary pattern. Serum vitamin D concentration was inversely associated with the prevalence of DM, HTN, and DL, but positively associated with the HLS. Among the HLS components, a balanced dietary pattern, regular physical activity, and alcohol consumption were positively associated with serum vitamin D concentration. Finally, the overall HLS was positively associated with serum vitamin D concentration.

Several epidemiological studies have demonstrated that a relatively low serum vitamin D concentration is associated with disturbed glucose metabolism and type 2 DM [21,22,23]. The biological mechanisms underlying these associations include pancreatic cell dysfunction and insulin resistance caused by defects in vitamin D-mediated calcium regulation [24].

Data on the association between HTN and vitamin D status remain inconclusive. Most, but not all, epidemiological studies have reported a significant association [25]. Plausible mechanisms underlying the association of serum vitamin D concentration with HTN include the upregulation of the renin–angiotensin–aldosterone system (RAAS), the inhibition of vascular smooth muscle cell proliferation, and insulin resistance [26]. Chronic vitamin D deficiency induces secondary hyperparathyroidism, which may stimulate the RAAS and elevate BP [27].

The association between DL and vitamin D status is ostensibly based on a biological mechanism related to vitamin D synthesis in the skin from 7-dehydrocholesterol, which is a cholesterol precursor, in the presence of ultraviolet-B radiation [28]. In fact, one meta-analysis on the effects of vitamin D supplementation on lipid parameters reported reduced blood levels of total cholesterol, low-density lipoprotein cholesterol, and triglycerides as well as elevated HDL cholesterol levels [29].

A higher prevalence of vitamin D deficiency has been observed in individuals with obesity [30]. This association may be explained by a genetic link between vitamin D and obesity, for example, the vitamin D receptor gene [31]. A meta-analysis of epidemiological studies reported a significant association between obesity and vitamin D deficiency [32]. Our results revealed a borderline association of BMI with serum vitamin D concentration; however, no association with obesity (defined as a BMI ≥ 25 kg/m^2^) was found, probably because of a lower BMI cutoff point for defining obesity. Future studies may need to explore more appropriate indicators of adiposity or use a prospective study design.

Smokers are reportedly more likely to have lower serum vitamin D levels [33]. As a biological mechanism underlying this association, smoking has been suggested to potentially inhibit vitamin D hydroxylation by decreasing serum parathyroid hormone levels [34]. In our study, no association was observed between smoking and vitamin D concentration. Because information on the duration of smoking was lacking in the KNHANES data, we were unable to evaluate lifetime smoking exposure (cigarette pack years). Further studies using more detailed information on smoking status are warranted to clarify its association with vitamin D status.

Data on the association between alcohol consumption and vitamin D status remain inconclusive. In a meta-analysis of 49 epidemiological studies [35], positive, inverse, and no associations were observed in 15, 18, and 16 studies, respectively. A previous epidemiological study analyzing KNHANES data yielded findings consistent with ours wherein increased alcohol consumption was associated with normal serum vitamin D levels in men [36]. In the current study, we confirmed a positive association between alcohol consumption and serum vitamin D concentration in a larger population size. Further studies are required to explore the biological mechanisms underlying this association, including gene expression related to alcohol metabolism.

Physical activity is reportedly associated with vitamin D status [37] partly because of sun exposure during outdoor activities. Notwithstanding, a low vitamin D concentration has been found to be related to physical functions such as gait speed and balance performance [38].

A previous study demonstrated that higher Mediterranean diet scores, which indicate a healthy diet, were significantly associated with a higher vitamin D concentration [16]. In another study, Iranian adults with healthy dietary patterns exhibited higher serum vitamin D levels than those with unhealthy dietary patterns [39]. Consistently, our study established a positive association of serum vitamin D concentration with a balanced dietary pattern, but not with an imbalanced dietary pattern. Among the HLS components, the healthy diet component showed a greater magnitude in the association with vitamin D concentration than others did in this study.

The strengths of this study include the analysis of national population-based survey data, the large sample size, the consideration of a broad range of potential confounding variables, and the comprehensive analysis of CVD risk factors. However, a causal inference could not be drawn owing to the cross-sectional nature of this study, posing a limitation to our findings. Furthermore, residual confounders caused by unmeasured variables might have existed; for example, the use of vitamin D supplements, hormonal status, such as estrogen and parathyroid hormone levels that may influence serum vitamin D concentration, and blood sampling seasonality. The generalization of our study’s results is limited by the specific characteristics of the study participants, who were middle-aged Koreans.

## 5. Conclusions

In summary, most CVD risk factors were found to be associated with serum vitamin D concentration. In particular, healthy lifestyle factors, such as a balanced diet and physical activity, were positively associated with a higher vitamin D concentration. Although further epidemiological studies are required to provide data on the causal inference of these associations, healthy lifestyle factors, which are generally considered to be CVD-protective factors, can be recommended for maintaining a healthy vitamin D status. In addition, future studies may need to explore biological mechanisms underlying the associations that we observed in this study.

## Figures and Tables

**Table 1:** Factor analysis results for dietary patterns.

Food Groups	Factor Loading Values ^1^	
	Imbalanced Diet	Balanced Diet
White rice	77 *	−2
Grains	−29	31 *
Potatoes/starch	−17	17
Noodles and dumplings	−25	3
Flour and bread	−26	30 *
Meat	13	32 *
Seaweeds	18	15
Seafood	24	32 *
Eggs	−4	37 *
Beans	12	30 *
Nuts	2	18
Vegetables	23	64 *
Kimchi	51 *	8
Mushrooms	3	25
Fruits	−17	32 *
Milk and dairy products	−40 *	26
Snacks and cakes	−11	9
Fast food	−12	−19

^1^ Presented as values multiplied by 100 and rounded to the nearest integer, and subsequently flagged with “*” when ≥30.

**Table 2:** Characteristics of the 11,703 participants according to serum 25-hydroxy vitamin D concentration quartiles.

Variables		Serum Vitamin D Concentration Quartiles [Median, ng/mL]				*p* Value
		1st [11.5]	2nd [15.7]	3rd [19.7]	4th [26.2]	
Age, years		49.46 ± 0.15	50.00 ± 0.16	50.67 ± 0.16	51.62 ± 0.16	<0.001
Sex	Male	35.6	46.2	56.6	62.8	<0.001
	Female	64.4	53.8	43.4	37.2	
Residential district	Cities	53.3	48.3	46.5	41.7	<0.001
	Other regions	46.7	51.7	53.5	58.3	
Household income	Lower-middle	37.7	36.6	37.9	38.6	0.688
level	High	62.3	63.4	62.1	61.4	
Body mass index, kg/m^2^		23.93 ± 0.08	24.24 ± 0.08	24.12 ± 0.07	24.01 ± 0.06	0.670
Educational level	Middle school graduate	32.1	34.2	35.8	43.5	<0.001
	Higher education	67.9	65.8	64.2	56.5	
Occupational type	Office work	21.3	22.8	21.1	16.5	<0.001
	Other	78.7	77.2	78.9	83.5	
Working hours	Full-time	31.5	35.2	34.8	30.5	0.002
	Other	68.5	64.8	65.2	69.5	
Sun exposure time	<5 h/day	80.2	78.6	75.8	64.9	<0.001
	≥5 h/day	9.8	13.4	17.0	27.2	
	No answer	10.0	8.0	7.2	7.9	
Sleep duration	<6 h/day	13.6	12.9	13.2	12.1	0.377
	6–7.9 h/day	58.9	60.8	59.2	58.1	
	8–9.9 h/day	24.8	23.8	25.4	26.8	
	≥10 h/day	2.7	2.5	2.2	3.0	
Calorie intake, kcal/day		1878.72 ± 17.22	2008.89 ± 18.18	2074.32 ± 19.09	2144.78 ± 19.31	<0.001
Use of dietary supplements		33.2	37.7	38.4	35.9	0.005
Physical activity	Regular waling	35.6	39.5	39.7	44.6	<0.001
	Moderate exercise	9.5	11.4	11.8	14.2	0.001
	Vigorous exercise	14.5	15.5	19.9	21.3	<0.001
Disease diagnosis	Diabetes mellitus	9.9	10.9	10.9	10.3	0.641
	Hypertension	28.6	28.2	28.1	30.8	0.174
	Dyslipidemia	38.4	39.9	42.3	39.6	0.096
Imbalanced diet factor scores		−0.04 ± 0.02	−0.05 ± 0.03	0.03 ± 0.02	0.13 ± 0.03	<0.001
Balanced diet factor scores		−0.17 ± 0.02	0.05 ± 0.02	0.13 ± 0.02	0.22 ± 0.02	<0.001
Healthy lifestyle components						
Healthy weight	BMI < 25 kg/m^2^	66.7	62.6	63.7	63.8	0.047
Physical activity ^1^		45.2	49.8	51.7	56.9	<0.001
Never smoked ^2^		64.6	56.0	49.3	43.7	<0.001
Alcohol drinking ^3^	1 or 2 drinks/day	86.9	81.0	78.7	76.3	<0.001
Balanced diet	Factor score > median	68.6	76.6	79.0	81.6	<0.001
Total healthy lifestyle score		3.32 ± 0.03	3.26 ± 0.02	3.22 ± 0.03	3.22 ± 0.03	0.003

Values are presented as the mean ± standard error or %. Statistical significance was evaluated using the χ^2^ or ANOVA test. ^1^ Participants who performed three physical activities (regular walking, moderate exercise, and vigorous exercise). ^2^ Participants who had smoked fewer than 100 cigarettes in their lifetime. ^3^ Calculated by multiplying the frequency of drinking by the quantity of a single drink; two drinks for men and one drink for women were defined as moderate alcohol drinking.

**Table 3:** Associations between cardiovascular risk factors and serum vitamin D concentration.

Variables		Number of Participants	Regression Coefficient Estimate (95% CI)		*p* Value for Trend
			Age- and Sex-Adjusted	Multiple-Adjusted ^1^	
Diagnosis of DM	No	10,457	Reference	Reference	
	Yes	1246	−0.691 (−1.140, −0.242)	−0.540 (−0.984, −0.096) *	
Diagnosis of HTN	No	8253	Reference	Reference	
	Yes	3450	−0.477 (−0.796, −0.158)	−0.342 (−0.654, −0.030) *	
Diagnosis of DL	No	7116	Reference	Reference	
	Yes	4587	−0.522 (−0.815, −0.230)	−0.494 (−0.784, −0.203) **	
BMI, kg/m^2^					0.102
<18.5		254	0.048 (−0.969, 1.065)	−0.007 (−1.010, 0.995)	
18.5–22.9		4246	Reference	Reference	
23.0–24.9		3077	0.183 (−0.188, 0.554)	0.270 (−0.092, 0.632)	
≥25.0		4126	0.056 (−0.273, 0.384)	0.261 (−0.073, 0.596)	
Balanced diet factor score					0.001
1st quartile		2925	Reference	Reference	
2nd quartile		2926	0.527 (0.146, 0.909)	0.486 (0.110, 0.861) *	
3rd quartile		2926	1.063 (0.688, 1.437)	1.017 (0.630, 1.404) ^†^	
4th quartile		2926	1.384 (0.982, 1.785)	1.292 (0.831, 1.753) ^†^	
Imbalanced diet factor score					0.163
1st quartile		2925	Reference	Reference	
2nd quartile		2926	−0.284 (−0.660, 0.092)	−0.178 (−0.549, 0.193)	
3rd quartile		2926	0.006 (−0.416, 0.427)	−0.021 (−0.430, 0.387)	
4th quartile		2926	0.641 (0.203, 1.079)	0.221 (−0.203, 0.644)	
Smoking status					0.083
Never smoked		7246	Reference	Reference	
Former smoker		2245	0.342 (−0.138, 0.822)	0.148 (−0.312, 0.607)	
Current smoker		2212	−0.147 (−0.646, 0.352)	−0.359 (−0.845, 0.127)	
Alcohol consumption					<0.001
Lifetime abstainer		1594	Reference	Reference	
Former drinker		3943	0.160 (−0.307, 0.627)	0.320 (−0.133, 0.773)	
Current smoker		6166	0.948 (0.475, 1.422)	1.085 (0.625, 1.545) ^†^	
Regular walking	No	6926	Reference	Reference	
	Yes	4774	0.755 (0.465, 1.045)	0.652 (0.371, 0.934) ^†^	
Moderate exercise	No	10,202	Reference	Reference	
	Yes	1501	1.215 (0.726, 1.704)	0.696 (0.225, 1.167) **	
Vigorous exercise	No	9695	Reference	Reference	
	Yes	2005	0.941 (0.569, 1.314)	0.614 (0.252, 0.976) **	

Abbreviations: CI, confidence interval; DM, diabetes mellitus; HTN, hypertension; DL, dyslipidemia. ^1^ Adjusted for survey year (continuous), age (continuous), sex, residential district (special cities, metropolitan cities, and rural areas), household income level (lower-middle and high), educational level (middle school graduate and higher education), occupational type (office, service, manufacturing, and not employed), working hours (full time, part time, and other), sun exposure time (<5 and ≥5 h/day and no answer), average sleep duration (<6, 6–7.9, 8–9.9, and ≥10 h/day), calorie intake (continuous), use of dietary supplements, regular physical activity, diagnosis of diabetes mellitus, hypertension, and dyslipidemia, body mass index (continuous), smoking status (never smoked, former and current smoker), alcohol consumption status (lifetime abstainer, former drinker, and current drinker), balanced diet factor scores (quartiles), and imbalanced diet factor scores (quartiles). * *p* < 0.05, ** *p* < 0.01, and ^†^ *p* < 0.001.

**Table 4:** Associations between healthy lifestyle scores and serum vitamin D concentration.

Variables		Number of Participants	Regression Coefficient Estimate (95% CI)		*p* Value for Trend
			Age- and Sex-Adjusted	Multiple Adjusted ^1^	
Healthy weight: <25 kg/m^2^		7577	0.022 (−0.257, 0.301)	−0.162 (−0.446, 0.122)	
Balanced diet: factor score > median		8778	0.979 (0.670, 1.289)	0.818 (0.498, 1.138) ^†^	
Smoking status: never smoked		7246	−0.080 (−0.517, 0.357)	−0.037 (−0.458, 0.385)	
Alcohol drinking: ≤ moderate		9865	−0.768 (−1.169, −0.367)	−0.629 (−1.022, −0.237) **	
Regular physical activity		6071	0.985 (0.700, 1.270)	0.774 (0.499, 1.049) ^†^	
Total HLS ^2^	0–1	524	Reference	Reference	0.002
	2	1777	0.451 (−0.264, 1.166)	0.433 (−0.266, 1.133)	
	3	3742	1.045 (0.389, 1.702)	0.914 (0.267, 1.562) **	
	4	4012	0.831 (0.181, 1.482)	0.653 (−0.004, 1.311)	
	5	1648	1.727 (0.997, 2.458)	1.405 (0.646, 2.165) **	

Abbreviations: CI, confidence interval. ^1^ Adjusted for survey year (continuous), age (continuous), sex, residential district (special cities, metropolitan cities, and rural areas), household income level (lower-middle and high), educational level (middle school graduate and higher education), occupational type (office, service, manufacturing, and not employed), working hours (full time, part time, and other), sun exposure time (<5 and ≥5 h/day and no answer), average sleep duration (<6, 6–7.9, 8–9.9, and ≥10 h/day), calorie intake (continuous), use of dietary supplements, diagnosis of diabetes mellitus, hypertension, and dyslipidemia, and body mass index (continuous; it was not adjusted for healthy weight). ^2^ Calculated based on five components (body mass index, a balanced diet, smoking status, alcohol drinking, and regular physical activity). ** *p* < 0.01, and ^†^ *p* < 0.001.

## Data Availability

All data are publicly available (https://knhanes.kdca.go.kr/knhanes/ (accessed on 4 September 2023)).
